# FAM83D promotes cell proliferation and motility by downregulating tumor suppressor gene FBXW7

**DOI:** 10.18632/oncotarget.1581

**Published:** 2013-11-25

**Authors:** Zeran Wang, Yueyong Liu, Pengju Zhang, Weiguo Zhang, Weijing Wang, Kenneth Curr, Guangwei Wei, Jian-Hua Mao

**Affiliations:** ^1^ Life Sciences Division, Lawrence Berkeley National Laboratory, One Cyclotron Road, Berkeley, CA, USA; ^2^ Department of Biology, California State University East Bay, Hayward, California, USA.; ^3^ Department of Biochemistry and Molecular Biology, Shandong University School of Medicine, Jinan, Shandong, China; ^4^ Department of Molecular and Cell Biology, University of California Berkeley, Berkeley, CA, USA; ^5^ Department of Anatomy, Shandong University School of Medicine, Building 1, Jinan, Shandong, P.R. China

**Keywords:** FAM83D, FBXW7, breast cancer, epithelial-mesenchymal transition, invasion

## Abstract

Abstract Amplification of chromosome 20q is frequently found in various types of human cancers, including breast cancer. The list of candidate oncogenes in 20q has expanded over the past decade. Here, we investigate whether FAM83D (family with sequence similarity 83, member D) on chromosome 20q plays any role in breast cancer development. The expression level of FAM83D is significantly elevated in breast cancer cell lines and primary human breast cancers. High expression levels of FAM83D are significantly associated with poor clinical outcome and distant metastasis in breast cancer patients. We show that ectopic expression of FAM83D in human mammary epithelial cells promotes cell proliferation, migration and invasion along with epithelial-mesenchymal transition (EMT). Ablation of FAM83D in breast cancer cells induces apoptosis and consequently inhibits cell proliferation and colony formation. Mechanistic studies reveal that overexpression of FAM83D downregulates FBXW7 expression levels through a physical interaction, which results in elevated protein levels of oncogenic substrates downstream to FBXW7, such as mTOR, whose inhibition by rapamycin can suppress FAM83D-induced cell migration and invasion. The results demonstrate that FAM83D has prognostic value for breast cancer patients and is a novel oncogene in breast cancer development that at least in part acts through mTOR hyper-activation by inhibiting FBXW7.

## INTRODUCTION

In recent years, extensive effort has been made towards a better understanding of the oncogenic events that drive breast cancer initiation and progression. However, successful treatments remain a challenge in many cases due to heterogeneity of breast tumors. Gene amplification is one of the most common genetic abnormality events in breast cancer. Chromosome 20q is frequently amplified in a variety of cancers including prostate, ovarian, bladder, colorectal, pancreatic, and breast [1-6]. Amplification of 20q is also associated with poor clinical outcome [7] and serves as an indicator for cancer progression and metastasis [6,8]. In breast cancer, multiple genes localized to 20q have been identified as oncogenes, including Aurora-A kinase (*AURKA*), *ZNF217* [9], *UBE2C* [10] and *TPX2* [11,12]. *AURKA* is the most studied gene on 20q. High expression levels of *AURKA* indicate decreased survival in breast cancer patients [13] and is currently an anticancer target [14]. Another gene on 20q, *ZNF217* was shown to be a marker for poor breast cancer prognostis [15,16] and its overexpression promotes epithelial-mesenchymal transition (EMT) and invasion [16]. However, the detailed and integral mechanism for how chromosome 20q affects tumorigenesis and tumor behavior is not clearly understood. Other genes on 20q are also likely to participate in tumorigenesis and/or metastasis, but their functions are yet to be defined.

Here we focus on the gene named ‘family with sequence similarity 83, member D’ (*FAM83D*), located on chromosome 20q. FAM83D was first identified as a protein with spindle localization in mitosis [17]. FAM83D interacts with the chromokinesin KID22 and is required for correct chromosome congression in metaphase [18]. The mitotic spindle is responsible for accurate distribution of sister chromatids in cell division. Functional aberration of the mitotic spindle may lead to errors in chromosome segregation and aneuploidy, which are often seen in advanced human cancers. Various microarray studies suggest that *FAM83D* expression is elevated in hepatoacellular carcinoma [19], ovarian cancer [20] and metastatic lung adenocarcinomas [21]. However, the function and mechanism of *FAM83D* in tumorigenesis has not yet been studied.

*FBXW7* is a bona fide tumor suppressor that is inactivated by gene mutation or expression downregulation in numerous human malignancies, including breast cancer [22]. It is a member of the F-box family of proteins, which function as the substrate recognition components of the Skp-Cullin-F-box (SCF) E3 ubiquitin ligase [22]. The SCFFBXW7 complex targets several well-known onco-proteins for ubiquitin-mediated degradation in a phosphorylation-dependent manner, including c-Jun, c-Myc, Cyclin E, KLF15, Notch and mTOR [23-28].

In the present study, we investigated whether *FAM83D* plays a role in breast cancer initiation and progression. We showed that overexpression of *FAM83D* inactivates *FBXW7* by downregulating FBXW7 protein expression, leading to up-regulation of FBXW7 downstream targets, which in turn results in elevated cell proliferation, migration and invasion.

## RESULTS

### Elevated expression of FAM83D in human breast cancers

**Figure 1:**
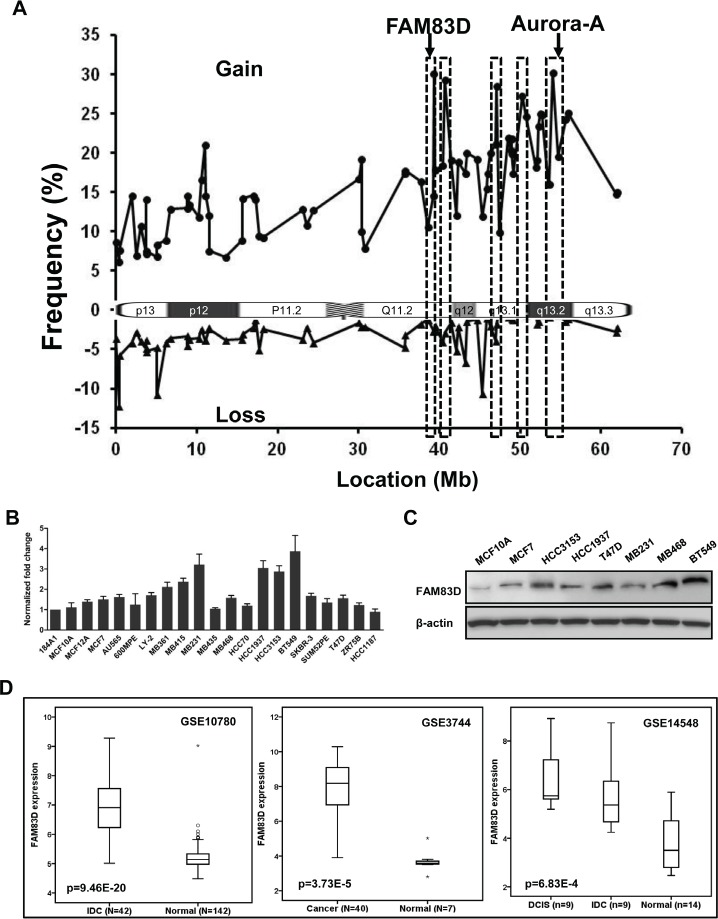
The expression of FAM83D is elevated in human breast cancers (A) Genomic amplification on chromosome 20q was refined by integrative analysis of public copy number datasets for breast cancers, indicating that *FAM83D* is located at a peak of a sub-amplicon. (B) Expression profile of *FAM83D* in breast cancer cell lines. *FAM83D* mRNA levels relative to normal breast epithelial cell line 184A1 were determined by qRT-PCR. Gene expression was normalized to GAPDH. Data are presented as means ± Standard deviation. (C) Protein level of FAM83D in cultured breast cancer cell lines. (D) *FAM83D* mRNA expression levels are significantly elevated in breast tumors in comparison to normal breast tissues, using three public expression datasets. *FAM83D* expression is measured as log_2_ (probe intensities). The p-values were obtained from Mann-Whitney U or Kruskal-Wallis tests.

We first revisited the CGH microarray data previously published on primary breast cancers [29-31] and cell lines [32] and refined 20q into 5 sub-amplicon regions, one containing *FAM83D* (Fig. 1A). Next we examined *FAM83D* expression levels in a panel of 20 widely used human breast cancer cell lines. As expected, we found that the level of *FAM83D* mRNA was elevated in most of the malignant cell lines by 1.5 to 4 fold, in comparison to levels in non-malignant cell lines MCF10A and 184A1 (Fig. 1B). Correspondingly, *FAM83D* protein levels are consistently increased in breast cancer cell lines (Fig. 1C). *FAM83D* expression was further assessed in three publicly available microarray datasets in the Gene Expression Omnibus (GEO) database (GSE10780 [33], GSE3744 [34], and GSE14548 [35]) that contain both normal and breast cancer samples. *FAM83D* expression levels were measured as log_2_ (probe intensities) using Affymetrix microarrays. In all three datasets, the levels of *FAM80D* mRNA in breast cancers were statistically significantly higher than those in normal breast tissues (Fig. 1D). These results indicate that the expression level of *FAM83D* is elevated in breast tumors.

### mRNA level of FAM83D is associated with clinical outcome of breast cancers

**Figure 2:**
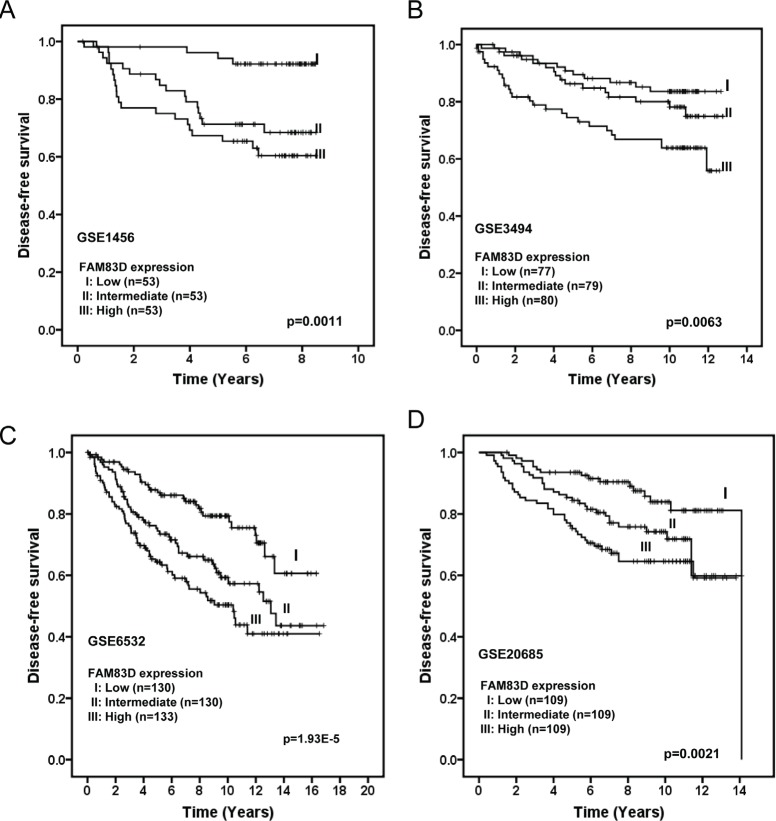
Elevated expression of FAM83D is associated with poor disease-free survival of breast cancer patients in four different cohorts (A) GSE1456, (B) GSE3494, (C) GSE6532, and (D) GSE20685 *FAM83D* expression is measured as log_2_ (probe intensities). The p-values were obtained from a long-rank test.

To investigate the clinical impact of elevated *FAM83D* expression in human breast cancer, we assessed the association between *FAM83D* mRNA levels and clinical outcome in four independent breast cancer cohorts [36-39] with clinical information (GEO database). To determine the prognostic impact of *FAM83D* expression in breast cancer, we categorized breast cancer patients into three groups based on *FAM83D* mRNA expression levels (low=bottom tertile, intermediate=middle tertile, and high=top tertile). Patients with tumors displaying high *FAM83D* expression levels had significantly shorter disease-free survival (DFS) compared to those with low *FAM83D* (p=0.0011, 0.0063, 1.93E-5 and 0.0021 in dataset GSE1456, GSE3494, GSE6532 and GSE20685, respectively) (Fig. 2). Moreover, in all four datasets, DFS curves for patients with intermediate levels of *FAM83D* was between those with high and low levels (Fig. 2), suggesting a dose-dependent effect of *FAM83D* expression on DFS.

### Overexpression of FAM83D promotes cell growth, migration and invasion

**Figure 3:**
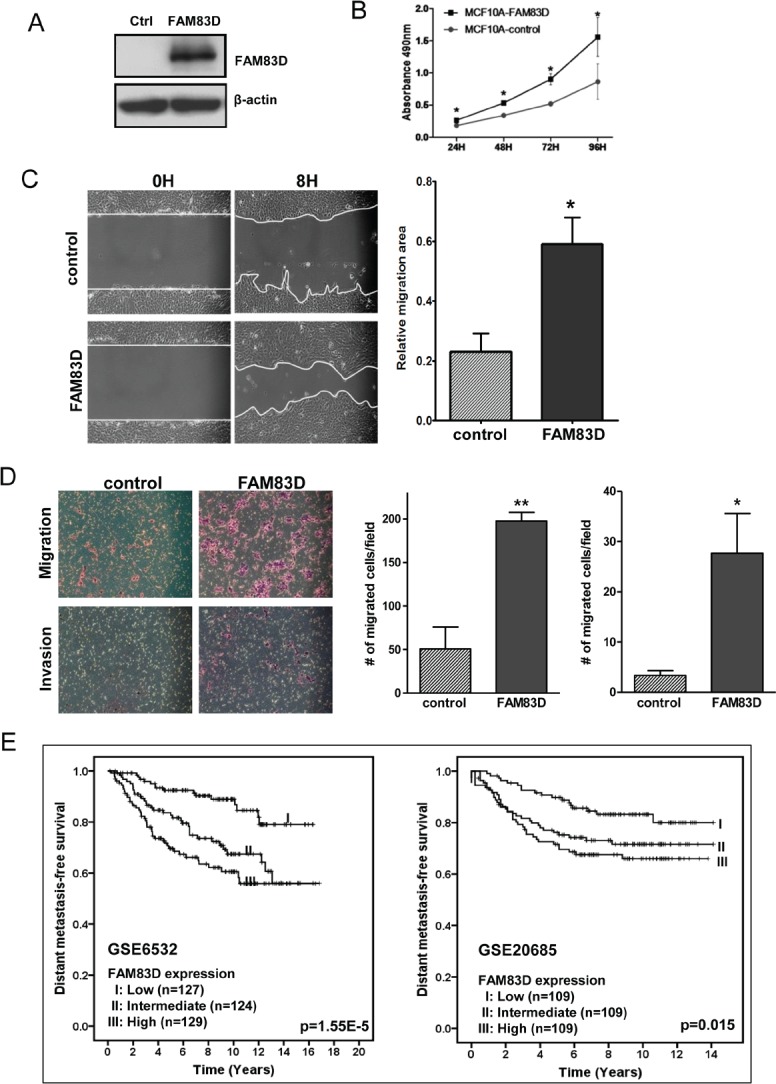
Ectopic expression of FAM83D enhances cell proliferation, migration and invasion (A) MCF10A cells with FLAG-tagged FAM83D (MCF10A-FAM83D) were established with stable expression of FAM83D and confirmed by Western blot using an anti-Flag antibody. (B) Proliferation of MCF10A-FAM83D is significantly accelerated compared to normal MCF10A control cells measured by MTT assay. (C) MCF10A-FAM83D (FAM83D) closed the scratched area significantly faster than MCF10A control cells (control). The representative photographs are shown in left panel. Quantification of the data is presented as relative migration area in each cell line (right panel). (D) Migration and invasion ability of MCF10A-FAM83D cells (FAM83D) is significantly enhanced compared to MCF10A control cells (control) using the transwell assay. The representative photographs are shown in the left panel and the average number of migrated cells/field in the right panel. In b-d, data are presented as means ± standard deviation from three independent experiments, each performed in triplicate. * indicates p < 0.05 and ** indicates p < 0.01, which were obtained by a t-test. (E) High *FAM83D* mRNA levels reduce distant metastasis-free survival for breast cancer patients in two independent datasets (GSE6532 and GSE20685). The p-values in the graphs were obtained from a long-rank test.

Since FAM83D is overexpressed in human tumors, we questioned whether FAM83D functions as an oncogene. To corroborate this hypothesis, we first tested whether overexpression of FAM83D in non-malignant MCF10A cells promotes cell growth. MCF10A cells have a low endogenous level of FAM83D (Fig. 1B). We generated MCF10A cells with stable expression of FLAG-tagged FAM83D gene (designated as MCF10A-FAM83D) (Fig. 3A). Overexpression of FAM83D strongly stimulates cell proliferation compared to control cells (Fig. 3B), indicating that FAM83D is potentially oncogenic.

Migration and invasion are hallmarks for cancer metastasis. To evaluate the functional role of FAM83D overexpression in cell motility in MCF10A cells, we performed an in vitro scratch healing assay and a Boyden chamber migration/invasion assay. As shown in Fig. 3C and 3D, FAM83D overexpression in MCF10A accelerated wound closure and migration through the uncoated membrane of the Boyden chamber compared to control cells. Moreover, MCF10A-FAM83D cells displayed a significant increase in the number of cells that migrated through the Matrigel-coated membrane in the Boyden chamber (invasion assay) (Fig. 3D). Consistent with these *in vitro* observations, we found that high level of FAM83D expression shortened the distant metastasis-free survival in breast cancer patients (Fig. 3E). Together, these results clearly suggest that FAM83D overexpression promotes cell motility.

### FAM83D induces epithelial–mesenchymal transition in MCF10A cells

**Figure 4:**
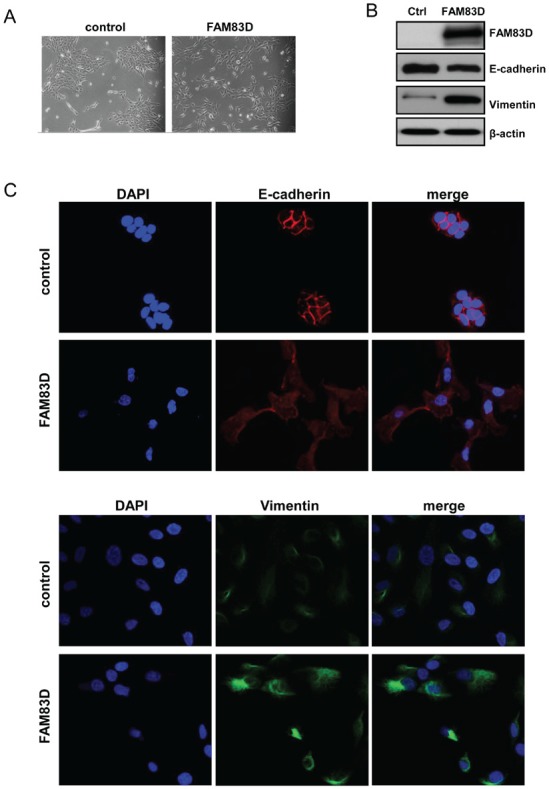
Overexpression of FAM83D in MCF10A cells leads to an epithelial-mesenchymal transition (A) Representative micrographs of MCF10A-FAM83D and MCF10A control cells under bright field display significant differences in morphology. (B) Western analysis of E-cadherin and vimentin indicates EMT in MCF10A-FAM83D compared to MCF10A control cells. Loading control is β-actin. (C) Immunofluorescent staining of E-cadherin (red, upper panels) and vimentin (green, lower panels) demonstrates EMT in MCF10A-FAM83D compared to MCF10A control cells. DNA is stained by DAPI (blue).

Given that ectopic expression of FAM83D increased cell motility and invasiveness, we next examined whether FAM83D overexpression could affect cell morphology. MCF10A-FAM83D cells exhibited a discohesive growth pattern and a spindle-shaped fibroblastic morphology, hallmarks of a mesenchymal phenotype, which was not observed in vector-only control cells (Fig. 4A). Consistently, the protein level of an epithelial marker E-cadherin is mildly suppressed in MCF10A-FAM83D cells (Fig. 4B). Moreover, immunofluorescent staining showed that the clear membrane distribution of E-cadherin MCF10A-control cells display, consistent with its normal function in cell-cell adhesion, was absent in MCF10A-FAM83D cells, and instead was enriched in the cytoplasm (Fig. 4C). Additionally, we found that Vimentin, a mesenchymal marker, dramatically increased in MCF10A-FAM83D cells (Fig. 4D). These findings suggest that FAM83D overexpression induces EMT.

### Ablation of FAM83D inhibits tumor growth through apoptosis

**Figure 5:**
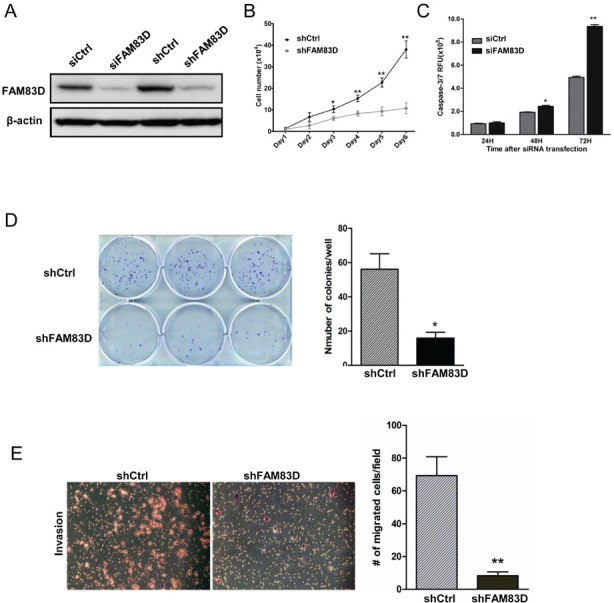
Biological consequences of FAM83D knockdown in BT549 cells (A) FAM83D expression was analyzed by Western blotting using anti-FAM83D antibody in both stable shRNA and transient siRNA knockdown in BT549 cells. Loading was controlled by β-actin levels. (B) BT549 cells with stable FM83D knockdown (shFAM83D) show a significant reduction in proliferation in comparison to control cells (shCtrl). BT549-shFAM83D and control cells were seeded in six-well dish in triplicates and cell numbers were counted by hemocytometer at indicated time points. (C) Knockdown of FAM83D induces cell apoptosis in BT549 cells. BT549 cells were transfected with siFAM83D or control siRNA (siCtrl) in a 96-well plate in triplicates for the indicated times. Apoptosis was determined by a Caspase3/7 assay. (D) Knockdown of FAM83D reduces colony formation in BT549 cells. (E) Knockdown of FAM83D significantly decreases cell invasiveness. Representative photographs from the Matrigel-coated transwell invasion assay of BT549 shCtrl control cells and shFAM83D cells. Data are presented as means ± standard deviation from three independent experiments, each performed in triplicate. * indicates p<0.05 and ** indicates p< 0.01, which were obtained from t-test.

Next we examined whether depletion of the high level of endogenous FAM83D proteins in BT549 cells (Fig. 1B) inhibited the cell proliferation and motility phenotypes. FAM83D was depleted using both stable (shFAM83D) and transient (siFAM83D) knockdown systems, which was verified at the protein level by Western blotting (Fig. 5A). Depletion of FAM83D dramatically inhibited BT549 cell proliferation (Fig. 5B). We observed many floating BT549-shFAM83D cells, implicating a potential apoptotic effect resulting from *FAM83D* depletion, which was confirmed by the Caspase3/7 apoptosis assay (Fig. 5C). Consistent with this result, we found a dramatic reduction in colony formation of BT549-shFAM83D cells (Fig. 5D). In addition, significantly fewer BT549-shFAM83D cells were able to migrate through Matrigel compared to controls (Fig. 5E), indicating that inhibition of FAM83D dramatically restrained the invasiveness of BT549 cells.

### FAM83D downregulates tumor suppressor FBXW7 via interaction

**Figure 6:**
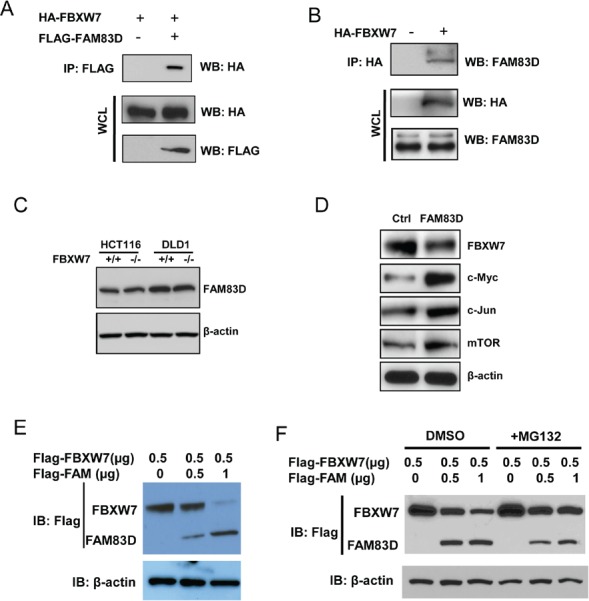
FAM83D downregulates FBXW7 via physical interaction (A) FLAG-FAM83D is specifically co-immunoprecipitated with HA-FBXW7. (B) Ectopic FBXW7 (HA-FBXW7) binds to endogenous FAM83D *in vivo*. (C) FAM83D protein levels are not regulated by FBXW7. (D) Overexpression of FAM83D in MCF10A represses FBXW7 expression and leads to elevated expression of known FBXW7 targets, including c-Myc, c-Jun and mTOR. (E) Ectopic overexpression of FAM83D (FLAG-FAM83D) reduces FLAG-FBXW7 protein levels in a dose-dependent manner. Amounts of transfected plasmids are indicated above the blot. (F) Proteasome inhibition by MG132 suppresses reduction of FLAG-FBXW7 by FAM83D ectopic overexpression. In co-immunoprecipitated experiments, 5% of extracts used were shown as loading controls (WCL). In Western-blotting experiments, β-actin serves as the loading control. In a and b, “-“ indicates empty vector.

To understand how FAM83D exerts its oncogenic effects, we used a yeast two-hybrid screening approach (data not shown) and discovered that FAM83D interacts with the F-box protein FBXW7. To confirm this result, 293T cells were co-transfected with FLAG-tagged FAM83D and HA-tagged FBXW7 constructs. Immunoprecipitation and subsequent immunoblot analyses revealed that HA-tagged FBXW7 co-immunoprecipitated with FLAG-tagged FAM83D, confirming a physical interaction between the two proteins (Fig. 6A). Moreover, we observed that HA-tagged FBXW7 co-immunoprecipitated with endogenous FAM83D (Fig. 6B), demonstrating that the physical interaction is not an artifact due to ectopic expression.

The physical interaction between FAM83D and FBXW7 suggested that FBXW7 could target FAM83D for ubiquitination and subsequent degradation. However, HCT116 and DLD1 FBXW7 knockout cells displayed no effect on FAM83D protein levels (Fig. 6C), implicating that FAM83D is not a downstream substrate of FBXW7.

We then addressed an alternative possibility in which FAM83D regulates FBXW7. Indeed, overexpression of FAM83D dramatically reduced endogenous FBXW7 protein levels (Fig. 6D), and was accompanied by increases in protein levels of FBXW7 substrates, including c-Myc, c-Jun and mTOR (Fig. 6D). Furthermore, FAM83D decreases the levels of FBXW7 proteins in a dose–dependent manner (Fig. 6E). Proteasome inhibition by addition of MG132 caused a significant increase in FBXW7 levels (Fig. 6F), suggesting that FAM83D promotes degradation of FBXW7.

### Inhibition of mTOR signaling suppresses FAM83D-induced oncogenic phenotypes

**Figure 7:**
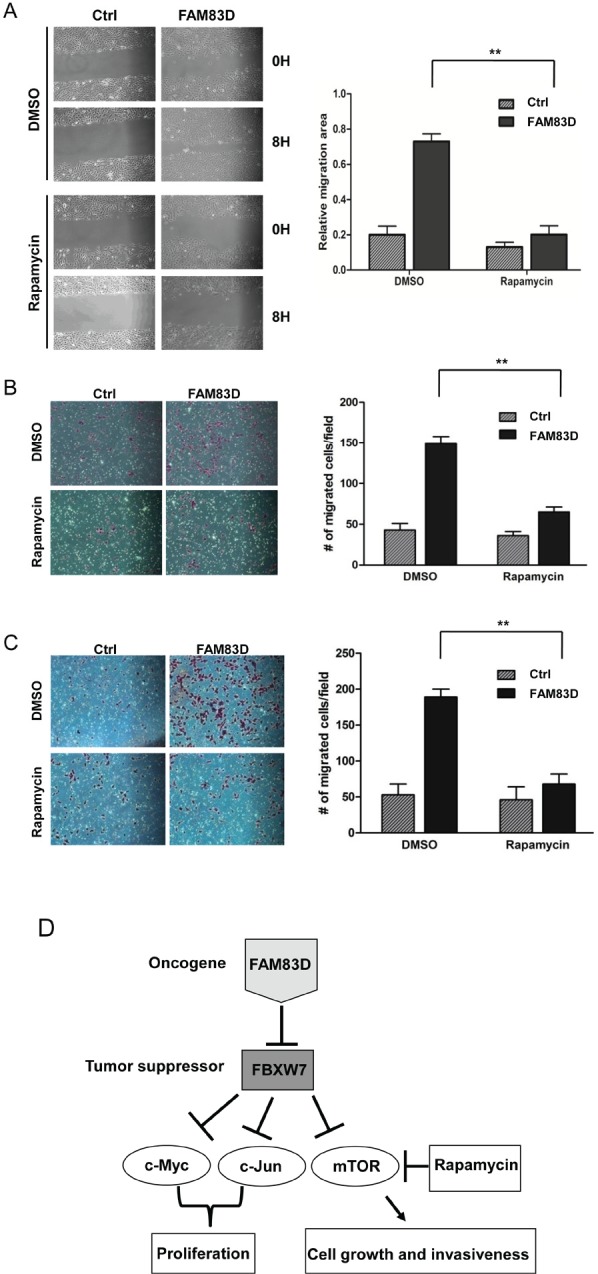
mTOR inhibition by rapamycin alleviates the enhanced migration and invasion caused by ectopic overexpression of FAM83D (A) Inhibition of mTOR by rapamycin significantly suppresses cell motility induced by FAM83D ectopic overexpression in scratch-healing assay. The representative photographs are shown in left panel. Quantification of the data was presented as relative migration area in each cell line (right panel). (B and C) Enhanced cell migration and invasion caused by FAM83D ectopic overexpression were rescued by treatment of Rapamycin. Uncoated (B) or Matrigel-coated (C) transwell assays on MCF10A control cells and MCF10A-FAM83D cells treated with DMSO control and rapamycin. In a-c, data are presented as means ± Standard deviation from three independent experiments each performed in triplicates. ** indicates p<0.01, which were obtained from t-test. (D) Schematic illustration of the proposed oncogenic function of FAM83D in breast cancer development through inhibition of the FBXW7 tumor suppressor by physical interaction.

Since FAM83D directly regulates FBXW7 to control cell growth and motility, we tested whether inhibition of downstream substrates of FBXW7 can suppress oncogenic phenotypes induced by FAM83D overexpression. We focused on mTOR, since our previous study showed that mTOR inhibition by rapamycin suppresses EMT, invasion and stemness driven by loss of FBXW7 in colon cancer cells [40]. As shown in Fig. 7A, rapamycin treatment significantly decreased the closure rate of MCF10A-FAM83D cells in the scratch healing assay. Consistent with this result, rapamycin also significantly decreased migration (Fig. 7B) and invasion (Fig. 7C) of MCF10A-FAM83D cells in the Boyden chamber assay. These results indicate that FAM83D executes oncogenic functions via regulation of FBXW7, which can be attenuated by inhibition of mTOR signaling, an FBXW7-downstream target.

## DISCUSSION

To our knowledge, this is the first study to report that *FAM83D* is an oncogene, based on several lines of evidence. First, we showed consistent overexpression of *FAM83D* mRNAs using a panel of breast cancer cell lines and primary breast cancers, and that high levels of FAM83D expression are associated with poor prognosis in breast cancer patients. Second, *in vitro* data provided direct evidence that *FAM83D* acts as an oncogene in breast cancer development by promoting cell growth, migration, and invasion. Third, overexpression of *FAM83D* induced EMT, a crucial mechanism regulating the initial steps in metastatic progression. Fourth, depletion of *FAM83D* induced cell death, reduced cell proliferation and colony formation, and abolished the invasive capability in aggressive and metastatic BT549 cells. Finally, we found that *FAM83D* downregulates the tumor suppressor gene *FBXW7*. These results led us to propose a model for how *FAM83D* executes its oncogenic function (Fig. 7D).

Mounting evidence shows that in epithelial cancers, including breast cancer, induction of EMT is a major event that provides mobility to cancer cells in order to generate metastases [41]. EMT is characterized by the loss of epithelial characteristics and acquisition of a mesenchymal phenotype, which confers the ability for cancer cells to invade adjacent tissue and migrate to distant sites [41]. In this study, we showed that ectopic expression of *FAM83D* in MCF10A cells displayed morphological and molecular characteristics in common with EMT, including the associated stimulatory effects on in vitro migration and invasion. These findings provide a mechanistic framework to explain the clinical observations that breast cancer patients with high levels of *FAM83D* in cancer tissue samples have significantly shorter disease-free survival. Silencing of *FAM83D* induced cell death, suggesting *FAM83D* is a potential target for cancer therapy to improve patients’ prognosis, especially for cancers with overexpression of *FAM83D*.

*FBXW7* is a tumor suppressor that controls the protein levels of many well-known oncogenes and is frequently mutated in many cancers [22]. However, little is known about the regulation of FBXW7 itself. We found that FAM83D physically interacts with and downregulates FBXW7 protein levels. Consequently, the levels of FBXW7-targeted oncoproteins, such as c-Myc, C-Jun and mTOR, are increased upon overexpression of FAM83D, which in turn leads to the increase in cell proliferation and motility. Surprisingly, inhibition of mTOR alone by rapamycin blocks *FAM83D*-induced oncogenic characteristics in cells, clearly suggesting that *FAM83D* executes its oncogenic function at least partially due to mTOR activation through FBXW7 downregulation. However, further experiments are necessary to explore the possible involvement of other oncogenic pathways.

Several mechanisms have been reported for inactivation of *FBXW7* in human cancer including mutation, deletion and hypermethylation [22, 42, 43]. A lot of effort has focused on finding *FBXW7* mutations in various types of human cancer, which have shown that the overall point mutation frequency is only 6% in human cancers [43]. In breast cancer especially, the somatic mutation rate of the FBXW7 gene was found to be less than 1%, based on The Catalogue of Somatic Mutations in Cancer Database (http://cancer.sanger.ac.uk/cancergenome/projects/cosmic/). Our study identifies an alternative mechanism for *FBXW7* inactivation in breast cancer through overexpression/amplification of *FAM83D*. This finding may be extended to a variety of other human cancers.

In summary, this study demonstrates that FAM83D promotes oncogenic properties upon overexpression, and physically interacts with FBXW7. Overexpression of FAM83D in tumor cells leads to downregulation of FBXW7, resulting in elevated levels of numerous oncoproteins downstream of FBXW7. Targeting the interaction between FAM83D and FBXW7 could serve as an effective cancer therapy.

## MATERIALS AND METHODS

### Cell lines and plasmids

The cell lines MCF10A and BT549 were maintained as previously described [32]. C20ORF129 (1758) cDNA was a gift from Dr. Erich A. Nigg. Full length FAM83D (1848bp) was generated by ligation of the 90bp fragment into 5' of *C20ORF129. FAM83D* was cloned into a 3XFLAG-HA tagged CMV10 vector, which was subsequently cloned into a pBabe.puro retroviral vector (Cell Biolabs).

### Knockdown of FAM83D by short hairpin RNA

The shRNA-expression vector was generated by cloning the annealed sense (GATCCCCatggacggatggcaaattaTTCAAGAG AtaatttgccatccgtccatTTTTTGGAAA) andantisense (AGCTTTTCCAAAAAatggacggatggcaaatta TCTCTTGA AtaatttgccatccgtccatGGG) oligos into the pSUPER.puro retroviral vector (Oligoengine). Viral particles were produced by transfection of the pSUPER vector into 293T ectopic Phoenix cells using Lipofectamine 2000 (Invitrogen). Stable shFAM83D BT549 clones were selected with puromycin (2ug/ml), and empty pSuper.Retro.puro vector was used as control.

### RNA isolation and real-time RT-PCR

RNA was isolated using the TRIzol Reagent (Invitrogen). First strand cDNA was synthesized with the SuperScript III (Invitrogen) or iScript (Bio-Rad) cDNA synthesis kits. All primers for qRT-PCR are: FAM83D, forward: 5'-CGCGAGAGGTGATTGCAGTGGT-3'; reverse: 5'-CGCGAGAGGTGATTGCAGTGGT-3'. GAPDH: forward: 5'-AAGGTGAAGGTCGGAGTCAA-3'; reverse: 5'-AATGAAGGGGTCATTGATGG-3'. qRT-PCR was performed using the Power SYBR Green PCR Master Mix (ABI) and the CFX96 Real-time PCR detection system (Bio-Rad), according to manufacturers’ protocols.

### Cell proliferation assay

Cells were seeded in 96-well plates in triplicate at densities of 1×10^3^ per well. Cell proliferation was monitored at desired time points using 3-(4, 5-dimethylthiazol-2-yl)-2, 5-diphenyltetrazolium bromide (MTT) (Promega). In brief, the MTT assay was performed by adding 20 μl MTT (5mg/ml) for 4 hrs. Light absorbance of the solution was measured at 570 nm on a microplate reader.

### Colony formation assay

Cells were seeded in triplicate at 500 cells/6-cm dishes in complete medium. After 3 weeks of growth, cells were fixed and stained with crystal violet (0.1%, w/v in 20nM 4-morpholinepropanesulfonic acid), and visible colonies were counted to determine cell numbers in each colony.

### Scratch-healing assay

Monolayers of MCF10A and MCF10A-FAM83D cells grown in 6-well plates were scratched by a sterile 200 µl pipette tip, washed 3 times with DPBS then cultured for the indicated time. Photographs focusing on the same position were taken immediately and after indicated time with a Nikon camera.

### Motility and invasion assay

2.5 x 10^5^ (invasion) or 2.5 x 10^4^ (motility) cells were resuspended in DMEM/F12 1:1 medium and seeded on BD Falcon Cell Culture Inserts with or without a thin layer of MATRIGEL Basement Membrane Matrix. The inserts were then placed on 6-well plates containing complete medium acting as chemo-attractant. After incubation for 16 hours, inserts were washed with PBS, fixed with 4% formalin (Sigma), permeabilized with methanol and stained with Giemsa staining solution (Invitrogen). The unmigrated cells on the surface of the membrane were removed using cotton swabs. Images were taken and analyzed with ImageJ to acquire cell numbers.

### Apoptosis Assay

BT549 cells were transfected with a negative control or siFAM83D RNA oligos (Ambion) for 24, 48 and 72 hours. Caspase 3/7 activity was measured at each time point with the Caspase-Glo 3/7 Assay kit (Promega) according to the manufacturer's instructions.

### Antibodies and Western blotting

Antibodies against E-cadherin and Vimentin, are from Cell Signaling, and anti-flag and anti-HA antibodies are from Sigma. For Western blots, cells were washed with cold PBS and lysed in RIPA lysis buffer (1% Triton X-100, 0.1% SDS, 50mM Tris PH 7.5, 150mM NaCl, 0.5% sodium deoxycholate, 10mM NaF) supplemented with 2 mM PMSF, 2 mM Na_3_VO_4_, and protease inhibitors (Roche). Cell lysates were fractionated on Novex® Tris-Glycine gels (Invitrogen) and transferred to PDVF membranes (Millipore) before antibody detection.

### Confocal immunofluorescence microscopy

Cells were seeded on glass coverslips in 4-well plates then fixed with 4% paraformaldehyde or methanol, then permeabilized for 5 min with PBS containing 0.1% Triton X-100. These cells were then blocked for 30min in 10% BSA and then incubated with primary antibodies overnight at 4°C. After three washes in PBS, the slides were incubated for 1 hour in the dark with FITC-conjugated secondary antibodies (Invitrogen, Grand Island, NY, USA). After three further washes, the slides were stained with DAPI for 5 min to visualize the nuclei, and examined using a Confocal Zeiss Axio Observer D1 microscope and images were analysis by ImageJ software.

### Statistical analysis

Differences in *FAM83D* mRNA expression levels between normal and breast cancers was analyzed by Mann-Whitney U (two groups) or Kruskal-Wallis (three groups) tests using publicly available datasets downloaded from the Gene Expression Omnibus (GEO). Kaplan-Meier plots were constructed and a long rank test was used to evaluate differences in *FAM83D* expression levels for disease-free and distant metastasis-free survival curves. In addition, the relationship between *FAM83D* expression and survival was explored in microarray datasets by dividing the cases from each cohort into a group with high (top one-third), moderate (middle one-third), and low (bottom one-third) levels of expression. All analyses were performed using SPSS 11.5.0 for Windows. A two-tailed p-value of less than 0.05 was considered to indicate statistical significance. We determined the significance of differences in cell proliferation, migration, invasion and apoptosis using two-tailed Student's t-test (MS EXCEL 2007 or Graphpad Prism 5). Real-time RT-PCR data analysis was performed using CFX96 Manager (Bio-Rad).
